# Essential role for STAT3/FOXM1/ATG7 signaling-dependent autophagy in resistance to Icotinib

**DOI:** 10.1186/s13046-022-02390-6

**Published:** 2022-06-11

**Authors:** Xin Lyu, Lizhong Zeng, Jie Shi, Zongjuan Ming, Wei Li, Boxuan Liu, Yang Chen, Bo Yuan, Ruiying Sun, Jingyan Yuan, Nannan Zhao, Xia Yang, Guoan Chen, Shuanying Yang

**Affiliations:** 1 Department of Pulmonary and Critical Care Medicine, Second Affiliated Hospital, Xi’an Jiaotong University, No. 157, Xiwu Road, Xincheng District, Xi’an, 710004 Shaanxi People’s Republic of China; 2 School of Medicine, Southern University of Science and Technology, No. 1088, Xueyuan Road, Nanshan District, Shenzhen, 518055 Guangdong China

**Keywords:** Non-small cell lung cancer, Icotinib, Resistance, Autophagy, ATG7

## Abstract

**Background:**

The contribution of autophagy to cancer therapy resistance remains complex, mainly owing to the discrepancy of autophagy mechanisms in different therapy. However, the potential mechanisms of autophagy-mediated resistance to icotinib have yet to be elucidated.

**Methods:**

The effect of autophagy in icotinib resistance was examined using a series of in vitro and in vivo assays. The results above were further verified in biopsy specimens of lung cancer patients before and after icotinib or gefitinib treatment.

**Results:**

Icotinib increased ATG3, ATG5, and ATG7 expression, but without affecting Beclin-1, VPS34 and ATBG14 levels in icotinib-resistant lung cancer cells. Autophagy blockade by 3-MA or silencing Beclin-1 had no effects on resistance to icotinib. CQ effectively restored lung cancer cell sensitivity to icotinib in vitro and in vivo. Notably, aberrantly activated STAT3 and highly expressed FOXM1 were required for autophagy induced by icotinib, without the involvement of AMPK/mTOR pathway in this process. Alterations of STAT3 activity using genetic and/or pharmacological methods effectively affected FOXM1 and ATG7 levels increased by icotinib, with altering autophagy and icotinib-mediated apoptosis in resistant cells. Furthermore, silencing FOXM1 impaired up-regulated ATG7 induced by STAT3-CA and icotinib. STAT3/FOXM1 signalling blockade also reversed resistance to icotinib in vivo. Finally, we found a negative correlation between STAT3/FOXM1/ATG7 signalling activity and epidermal growth factor receptor-tyrosine kinase inhibitors (EGFR-TKIs) treatment efficacy in patients undergoing EGFR-TKIs treatment.

**Conclusions:**

Our findings support that STAT3/FOXM1/ATG7 signalling-induced autophagy is a novel mechanism of resistance to icotinib, and provide insights into potential clinical values of ATG7-dependent autophagy in icotinib treatment.

**Graphical abstract:**

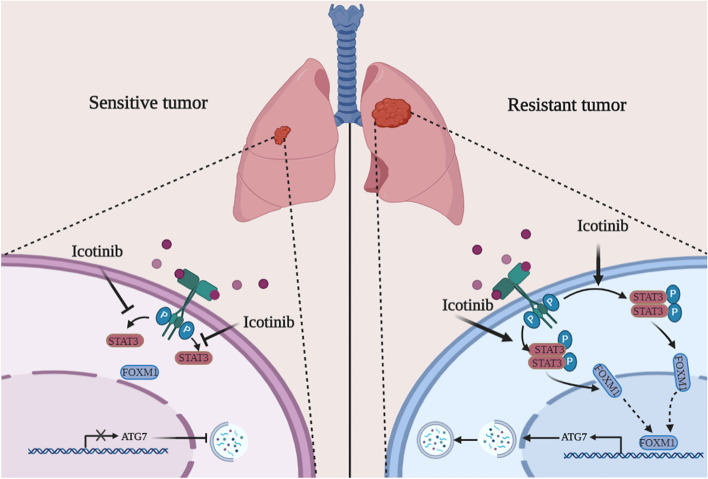

**Supplementary Information:**

The online version contains supplementary material available at 10.1186/s13046-022-02390-6.

## Background

Icotinib has shown a favourable efficacy and appeared to be well tolerated in NSCLC patients harbouring EGFR mutations. Previous clinical trials reported that icotinib could prolong progression-free survival (PFS) and overall survival (OS) of patients with EGFR exon 19 deletion or L858R mutation [1]. Unfortunately, partial patients with wild-type EGFR NSCLC could not benefit from the initial treatment with icotinib [2], and other patients with EGFR mutant NSCLC only received short-term clinical benefits from icotinib treatment [3], indicating that innate and acquired resistance limit broad application and long-term efficacy of icotinib. Thus, it is required to clarify the possible resistance mechanisms and explore corresponding treatment strategies.

Autophagy has been considered as a highly conserved and complex process whereby sequential formation of autophagosomes and autolysosomes, and component degradation [4]. As an essential protein in autophagy activation, ATG7 appears to play a scenario-dependent role in the cross-talk between autophagy-dependent resistance and autophagic apoptosis. For instance, ATG7-dependent autophagy facilitated promoting erlortinib resistance and silencing ATG7 overcame resistance of breast cancer cells to erlortinib [5]. On the contrary, ATG7 was also required for autophagic apoptosis under the treatment of erlortinib in lung cancer with EGFRT790M [6]. Thus, the role and mechanisms of ATG7 in icotinib resistance need to be explored.

The JAK2/STAT3 pathway and PI3K/AKT/FOXM1 pathway have been identified as primary targets of EGFR-TKIs. Of note, activation of STAT3 and FOXM1 diminished the therapeutic advantages of EGFR-TKIs [7, 8]. On the other hand, STAT3 and FOXM1 are also considered as upstream molecules in autophagy. They inhibited autophagy by preventing the formation of Beclin-1/VPS34 complex [9], and rendered tumour cells resistant to chemotherapy by targeting AMPK/mTOR pathway-induced autophagy [10]. Nevertheless, it is worth exploring whether and how STAT3 and FOXM1 affect icotinib resistance and autophagy.

Based on the above observations, we demonstrated the responses of EGFR-mutated NSCLC cells to icotinib, and explored the biological functions and action mechanisms of autophagy in icotinib-resistant cells in vitro and in vivo. Further study will be needed to verify the relationships between autophagy and EGFR-TKIs resistance in EGFR-mutated NSCLC tissues after EGFR-TKIs treatment.

## Methods

### Cell culture, treatments and reagents

Human cancer cell line PC-9 was purchased from the American Type Culture Collection (ATCC, Manassas, VA, USA) and genotyped for identity at China Center for Type Culture Collection (CCTCC, Wuhan, China). Gefitinib-resistant PC-9/GR cells were gifted by Prof. Shiyue Li and Dr. Ming Liu from Guangzhou Medical University (Guangzhou, China). H1975 cells were kindly provided by Dr. Xinling Ren from Air Force Military Medical University (Xi’an, China). These cell lines were all cultured with DMEM or RPMI 1640 medium (HyClone, Utah, USA) supplemented with 10% fetal bovine serum (FBS, BI, Israel) at 37C, with 5% CO2. To prepare for further studies, PC-9/GR and H1975 cells were exposed to 2 μM icotinib or DMSO for 4 weeks. Icotinib was provided by Betta Pharmaceuticals Co., Ltd. (Zhengjiang, China). 3-MA, bafilomycin A1 (BafA1), and chloroquine (CQ) were purchased from Sigma Aldrich (Merck KGaA, Darmstadt, Germany). Cryptotanshinon (CTN) was obtained from MCE (USA).

### Cell viability assay

Cell viability was assessed with Cell Counting Kit (CCK)-8 (Dojindo Molecular Technologies, Kumamoto, Japan). Lung cancer cells were seeded into a 96-well plate at a density of 5 × 103/well, and treated with various concentrations of icotinib for up to 24 h, 48 h and 72 h. After treatment, 20ul of CCK-8 was added to the cells and the incubation was continued for 2 h. Cell viability was measured by the absorbance at 450 nm on an ELISA reader (Bio-Rad Laboratories, Hercules, CA, USA).

### Cell apoptosis and clonogenic assays

Procedures of cell apoptosis and clonogenic assays were performed as described previously [11]. Cancer cells were seeded into a 6-well plate of 3 × 105/well, and harvested by a set of treatment, trypsinization, centrifugation, wash, and staining with Annexin V-PE and 7-AAD. Apoptosis was detected by flow cytometry (Becton-Dickinson; BD Biosciences). For clonogenic assay, cells were seeded in 6-well plates at a density of 250–300 cells/well, and treated with the indicated treatment conditions. Cells were then fixed with 4% PFA (Servicebio Technology CO., LTD, Wuhan, China), stained with crystal violet (Solarbio, Beijing, China) and photographed using a camera (Canon, Tokyo, Japan).

### Transmission Electron microscopy (TEM)

Cells and tissues were fixed with the electron microscope fixative (Servicebio Techology CO., LTD, Wuhan, China) overnight. After osmium acid fixation and dehydration, the samples were embedded to prepare ultrathin sections. The intracellular structures were detected using TEM HT7700 (Hitachi, Tokyo, Japan).

### Autophagic flux experiments measurement

The mRFP-GFP-LC3 lentivirus vectors were used to monitor autophagy flux (GeneChem Biotechnology Co., Ltd., Shanghai, China). According to the manufacturer’s instructions, lung cancer cells were infected with mRFP-GFP-LC3 lentivirus. Following an incubation period of 8 h, cells were replaced with complete medium for 48 h. Puromycin was added into cells for 1 week, and puromycin resistant cells were survived. After treatment, fluorescence images were observed by laser confocal fluorescence microscopy IX83 (Olympus, Tokyo, Japan). In pictures, yellow dots represent autophagosomes and red dots represent autolysosomes, which were counted in at least 100 cells in each sample.

### Short interfering RNA (siRNA) and plasmids transfection

SiRNAs targeting ATG3, ATG7, Beclin-1 and negative control were purchased from Santa Cruze Biotechnology (Dallas, Texas, USA). SiRNAs targeting FOXM1 (siFOXM1-1: 5′-CUCUUCUCCCUCAGAUAUA-3′, siFOXM1-2: 5′-GCCGGAACAUGACCAUCAA-3′ and control siRNA (si-NC 5′-TAAGGCTATGAAGAGATAC-3′) were synthesized by GenePharma (Shanghai, China). Wild-type STAT3 plasmids (pGV492-Flag-STAT3WT), constitutively active STAT3 plasmid (pGV492-Flag-STAT3-CA), dominant-negative STAT3 vector (pGV492-Flag-STAT3-Y705F) or the respective empty vectors plasmids were constructed by GeneChem. After exposed to DMSO or icotinib for the indicated time, cancer cells were seeded into a 6-well plate and transfected with siRNAs or plasmids for 72 h using RNAiMAX or Lipofectamine2000 (Invitrogen, MA, USA). Then cells were collected as indicated.

### Immunoblotting, immunohistochemistry (IHC) and TUNEL staining

For immunoblotting, total protein in lung cancer cells and xenografted tumour tissue was harvested with RIPA lysis buffer (Beyotime, Shanghai, China) containing a protease/phosphatase inhibitor cocktail (MCE, USA). Following procedures were performed as previously described [11]. For IHC, Paraffin-embedded sections of LUAD specimens and xenografts were subjected to heat-induced epitope retrieval and blocked with 3%H2O2 and goat serum at room temperature. After incubation with primary antibodies and secondary antibodies linked with DAB, images were captured and scored using the IRS (immunoreactive score, IRS) score system based on the intensity and percentage of positive staining [12]. All slides were viewed and assessed by two independent pathologists. For TUNEL staining, apoptotic Cells in xenograft tissues were identified by the TdT-mediated dUTP labelled by fluorescein after retrieval and permeabilization, and the nucleus was stained with DAPI. Photograph acquisition was finished using a fluorescence microscope (Olympus, Tokyo, Japan).

### Plasmid and shRNA Lentivirus infection

Wild-type STAT3 plasmid, dominant-negative STAT3 plasmid (pGV492-Flag-STAT3-Y705F), FOXM1 shRNA (shFOXM1) and scrambled shRNA (shNC) lentivirus were purchased from Hanheng Biotechnology (Hanheng Biotechnology Co., Ltd., Shanghai, China). Cells were plated into six-well plates and infected with plasmids and shRNA lentivirus according to the manufacturer’s instructions. Six hours later, cells were washed with PBS and cultured with complete medium in the incubator for 48 h.

### Xenograft studies

All experiments involving animals were performed following the protocol of the National Institutes of Health Guide for the Care and Use of Laboratory Animals (NIH Publications No. 8023, revised 1978), and approved by the Laboratory Animal Center of Xi’an Jiaotong University. 4-5 weeks old female nude mice were purchased from Beijing Vital River Laboratory Animal Technology Co., Ltd. (Beijing, China), and kept in specific pathogen-free (SPF) rooms at the Animal Center of Xi’an Jiaotong University for feeding and observations. Notably, to relieve the pain to these mice, a system containing isoflurane was used to introduce inhalation anaesthesia before the subcutaneous injections. The mice were sterilised with medical iodophors before and after subcutaneous injection. PC-9/GR cells (5 × 10^6^) were inoculated into the subcutaneous tissue of the right flanks of nude mice, and growth was observed every three days. To explore the impacts of CQ on resistance to icotinib in vivo, the mice were randomly divided into four groups of four mice each with or without oral administration of icotinib (50 mg/kg), or subcutaneous injection of chloroquine (50 mg/kg), or the indicated combinations for 21 days. To explore the role of STAT3/FOXM1 in resistance to icotinib in vivo, seven groups were designed: (1) Ctrl, (2) icotinib(50 mg/kg), (3) icotinib(50 mg/kg) plus LV-STAT3WT, (4) icotinib(50 mg/kg) plus LV-STAT3-Y705F, (5) icotinib(50 mg/kg) plus shNC, (6) icotinib(50 mg/kg) plus shFOXM1, (7) icotinib(50 mg/kg) plus LV-STAT3-Y705F and shFOXM1. When tumour volume reached to 50-100 mm^3^ in Ctrl group, the mice received drug treatment. At the endpoint of treatment, nude mice were sacrificed. Tumour volumes were measured as (length × width2) × 0.5. Tumour weight and body weight were evaluated at the endpoint of treatment and every three days, respectively.

### Patient-derived samples

Advanced lung adenocarcinoma patients detected for EGFR mutations at the 2nd Affiliated Hospital of Xi’an Jiaotong University from 2013 to 2018 were recruited in this study. Thirty-two biopsy specimens from patients were obtained before single-agent gefitinib or icotinib treatment, and the other thirty-one specimens were from patients who had developed radiographic progression of disease after continuous gefitinib or icotinib therapy. Biopsy specimens were obtained in the least invasive manners, including fine-needle aspiration (FNA) and core biopsy done with image guidance. The Ethics Committee of the 2nd Affiliated Hospital of Xi’an Jiaotong University approved this study. Before the study, all patients signed informed consent. The study was carried out in compliance with the Code of Ethics of the World Medical Association (Declaration of Helsinki Declaration of 2013).

### Statistical analysis

Comparisons among multiple groups were analyzed using the one-way ANOVA, and Student t-test was conducted for data between two groups. The correlation between EGFR-TKIs resistance and gene expression was examined using the Chi-square test. The rank-based Spearman correlations were conducted to detect the association among p-STAT3 expression, FOXM1 expression, and ATG7 expression. The Kaplan-Meier method and log-rank test were used to evaluate survival. A Cox proportional hazard regression model was used to evaluate the predictive value of STAT3/FOXM1/ATG7 signalling on the therapeutic effect of icotinib. Each assay in this study was carried out in triplicate, and all data were shown as mean ± standard deviation (SD). SPSS 23.0 (SPSS, Inc., IL, USA) and GraphPad Prism 7.0 (GraphPad Software, Inc., CA, USA) were separately used to perform statistical analysis and generate the set of graphics. *P* < 0.05 was considered statistically significant.

## Results

### The sensitivity/resistance to icotinib is capitulated in EGFR-mutated NSCLC cell lines

**Fig. 1:**
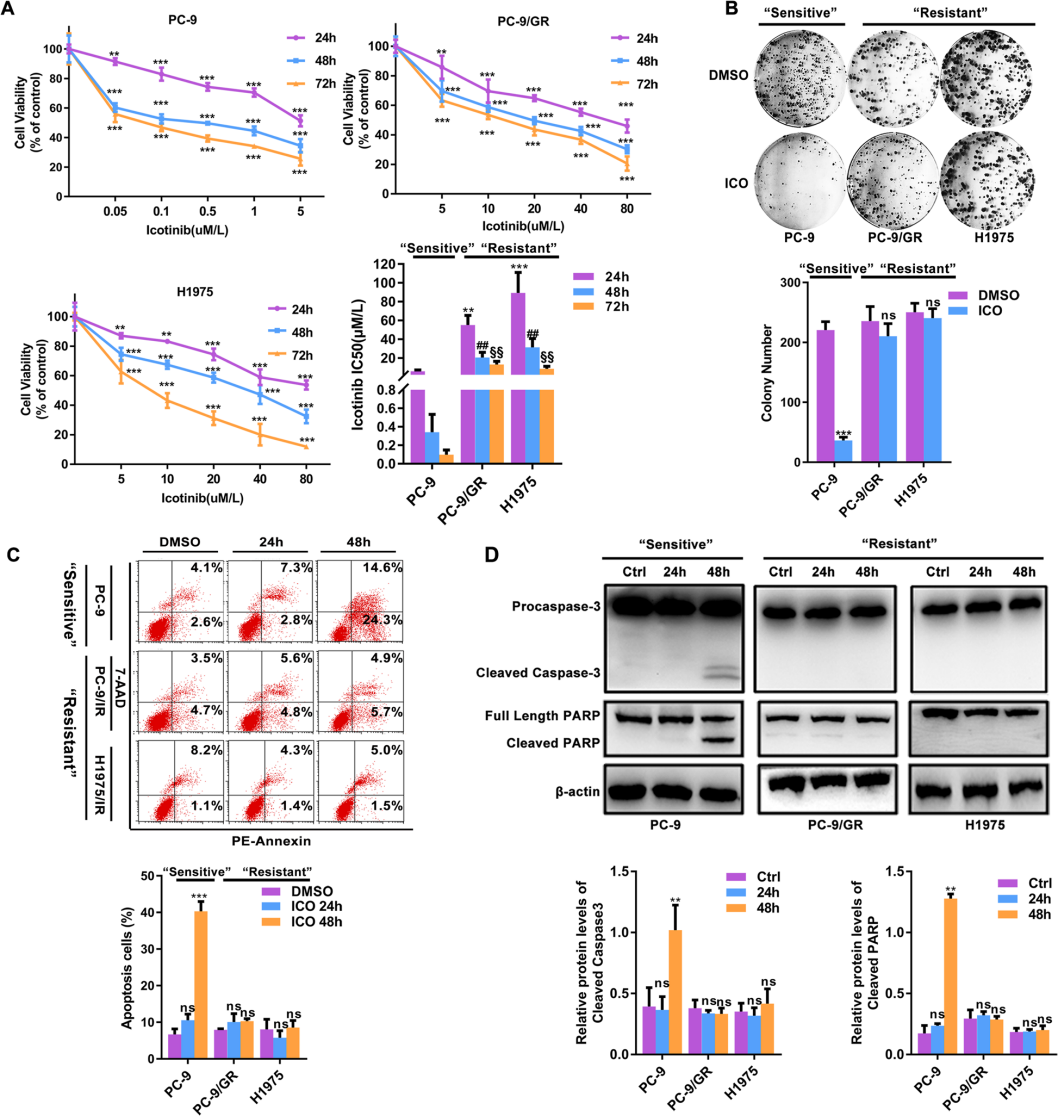
A subset of EGFR-mutated NCLC cells is highly resistant to icotinib. **A** Growth-inhibitory effects of indicated time and doses of icotinib were determined via CCK-8 assays, IC50s are depicted. **B** Clonogenic assays detected the response of EGFR-mutated NSCLC cells to icotinib. **C** Cell apoptosis induced by icotinib was displayed by flow cytometry analysis (Annexin V-7-AAD staining in the x-axis and PI in the y-axis). **D** Cleaved Caspase-3 and Cleaved PARP known as the markers of apoptosis were measured using immunoblotting assays. [In line charts, **P < 0.01, ***P < 0.001 as compared with the control group. In bar charts, **P < 0.01, ***P < 0.001 as compared with the 24 h group, ##*P* < 0.01vs. the 48 h group, §§ *P* < 0.001 as compared with the 72 h group]

The effects on cell viability and clonogenic capacity by icotinib were detected in EGFR- mutated lung cancer. With the dedicated treatment conditions, icotinib exhibited a time- and dose-dependent growth inhibition in lung cancer cell lines. Based on the differences of IC50s in distinct time, PC-9 was sensitive to low-dose icotinib, while similar responses in PC-9/GR and H1975 were dependent on high-dose icotinib above the clinically relevant dose (2 μM) (Fig. 1A). Similarly, this pattern was also reflected in the effects of icotinib on clonogenicity (Fig. 1B) and induction of apoptosis (Fig. 1C). Dramatic growth reduction and cell apoptosis were induced in the “sensitive” PC-9 cell line, while no significant changes in growth and apoptosis were seen in “resistant” PC-9/GR and H1975 cell lines. The expression of cleaved caspase3 and PARP were increased in “sensitive” but not in “resistant” cell lines (Fig. 1D). These data support that PC-9 is markedly sensitive to icotinib, while PC-9/GR and H1975 are resistant to icotinib.

### Class III PI3K complex-independent of autophagy mediates icotinib resistance in EGFR-mutated NSCLC cell lines

**Fig. 2:**
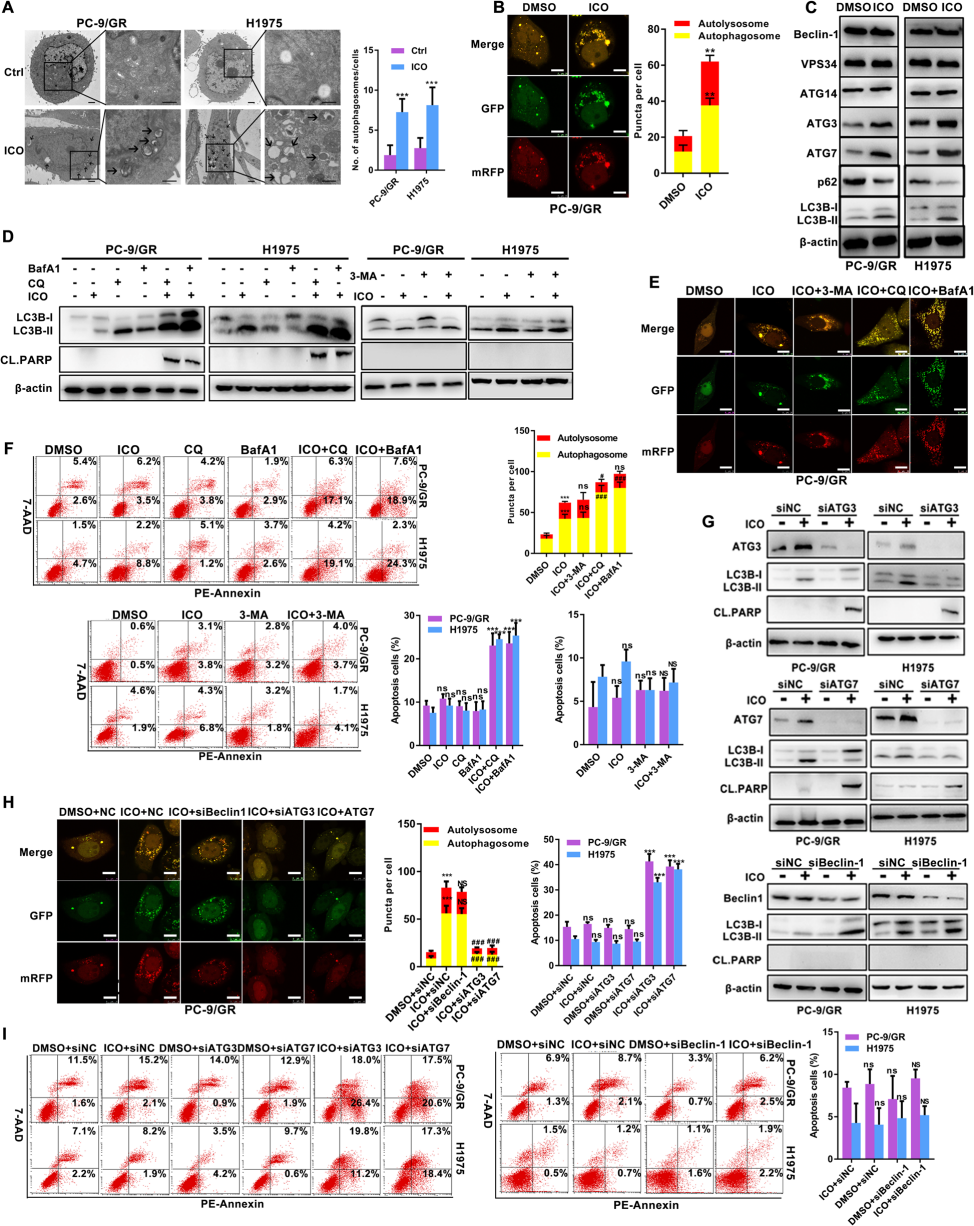
Protective autophagy induced by icotinib mediates icotinib resistance in EGFR-mutated NSCLC cells. **A** TEM depicted ultrastructural changes in NSCLC cells in response to icotinib (2 μM/4 weeks). Arrows point to autophagic vehicles. Scale bar: 1 μm. **B** PC-9/GR cells stably expressed mRFP-GFP-LC3 were treated with DMSO or 2 μM icotinib for 4 weeks, and were detected by laser confocal fluorescence microscopy. In the merged image, yellow puncta indicate autophagosomes, while red puncta indicate autolysosomes. Scale bar: 10 μm. **C** Immunoblotting assays assessed the expression of Beclin-1, VPS34, ATG14, ATG3, ATG7, LC3B and p62 in PC-9/GR and H1975 cells treated with icotinib or DMSO for 4 weeks. **D**-**F** Autophagy inhibitors CQ (10 μM/24 h), BafA1 (10 nM/24 h) and 3-MA (1 mM/24 h) were added to icotinib (2 μM/4 weeks) -exposed cells for 24 h. Autophagy analysis including immunoblotting detection of changes in cleaved PARP and LC3B conversion (**D**), and observations of mRFP-GFP-LC3 puncta (**E**) were observed in indicated cells. Scale bar: 10 μm. Cell apoptosis was analyzed using flow cytometry analysis (**F**). **G**-**I** Icotinib-exposed PC-9/GR and H1975 cells were transfected with siATG3, siATG7 and siBeclin-1. Autophagy analysis including immunoblotting detection of changes in cleaved PARP and LC3B conversion (**G**), and observations of mRFP-GFP-LC3 puncta (**H**) were observed in indicated cells. Scale bar: 10 μm. Cell apoptosis was analyzed using flow cytometry analysis (**I**). [**P < 0.01, ***P < 0.001, ns, no significance as compared with the control group. ###*P* < 0.001, NS, no significance as compared with the icotinib alone group]

To further explore the reasons for icotinib resistance, we used TEM to examine structural changes of “resistant” PC-9/GR and H1975 cell lines. After continuous exposure to icotinib for 4 weeks, a number of autophagic vesicles are observed in Fig. 2A. Furthermore, PC-9/GR cells exposed to icotinib exhibited increased autophagy flux, including yellow and red puncta (Fig. 2B). Notably, icotinib induced LC3B conversion but decreased the levels of p62 expression in resistant cells. Meanwhile, an increase in the expression of ATG3 and ATG7 but no effects on the expression of Beclin-1, VPS34, ATG14 were also observed in the group of icotinib (Fig. 2C). When icotinib was combined with autophagy inhibitors CQ or BafA1, both LC3B conversion and cleaved PARP expression induced by icotinib were further enhanced, the similar pattern was not displayed in the group of 3-MA and icotinib (Fig. 2D). Icotinib caused an increase of autophagy flux after adding CQ or BafA1 in “resistant” cells, but without apparent changes in the combination of icotinib and 3-MA (Fig. 2E). Furthermore, icotinib induced apoptosis and reduced clonogenic capacity in the addition of CQ and BafA1, but not in the presence of 3-MA (Fig. 2F, and Supplementary Fig. 1A). Silencing ATG3 and ATG7 decreased icotinib–induced LC3B-II expression but increased cleaved PARP levels. However, such alterations were not detected in the combination of icotinib and siBeclin-1 (Fig. 2G). The similar pattern of results appeared in the detection of autophagy flux (Fig. 2H). Meanwhile, enhanced apoptosis in response to icotinib was found after silencing ATG3 and ATG7, but the percentage of apoptotic cells was not altered by icotinib combined with siBeclin-1 (Fig. 2I). These data suggest that autophagy initiated by icotinib is irrelevant to the formation of class III PI3K complex.

### STAT3 is essential for icotinib-initiated protective autophagy

**Fig. 3:**
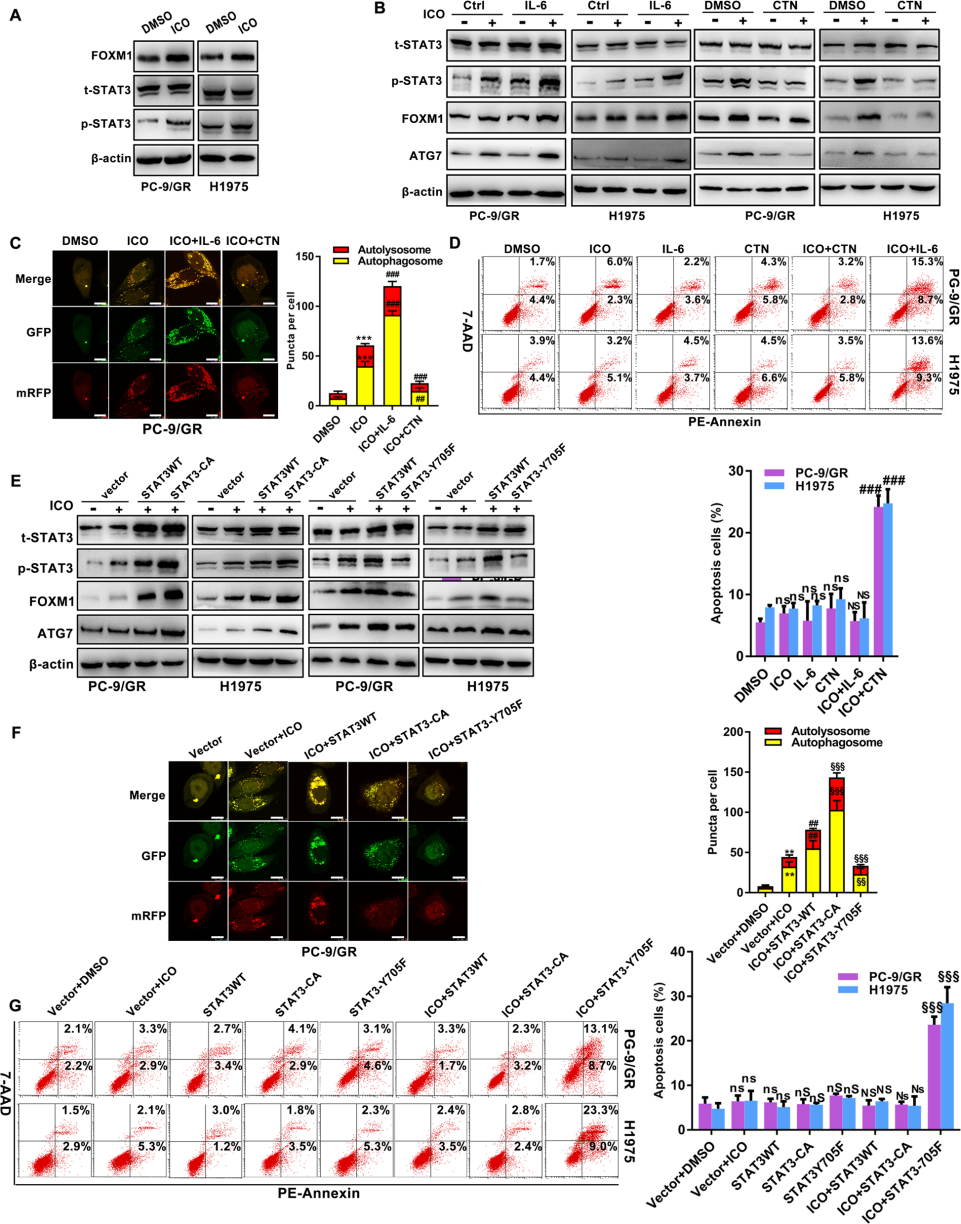
STAT3 regulates ATG7-dependent autophagy in icotinib resistant cells. **A** Immunoblotting analysis of changes induced by icotinib in STAT3 activation and FOXM1 levels in indicated cells. **B**-**D** Detection of protein changes in STAT3 activity, FOXM1 and ATG7 levels (**B**), autophagic flux with the mRFP-GFP-LC3 reporter (**C**), and cell apoptosis (**D**), induced by icotinib in indicated cells with or without the treatments of IL-6 (10 ng/ml/24 h) or CTN (7 μM/24 h). **E**-**G** In the indicated cells with or without icotinib treatment, protein changes in STAT3 activation, FOXM1 and ATG7 levels (**E**), the autophagic flux with the mRFP-GFP-LC3 reporter (**F**), and apoptosis (**G**) in the absence and presence of STAT3WT, STAT3-CA or STAT3-Y705F plasmids, are shown. [**P < 0.01, ***P < 0.001, ns, no significance as compared with the DMSO group. ##*P* < 0.01, ###*P* < 0.001, NS, no significance as compared with icotinib group. §§*P* < 0.01, §§§*P* < 0.001, Ns, no significance as compared with the combination group of icotinib and STAT3WT. nS as compared with the group of STAT3WT]

Although STAT3 was the therapeutic target of icotinb, it is unclear whether it triggered autophagy in icotinib resistance. To that end, we attempted to test the potential impacts of STAT3 in icotinb-induced autophagy using pharmacological and genetic methods. Icotinib up-regulated the expression of p-STAT3 and FOXM1, without affecting the expression of STAT3 (Fig. 3A). Then we cultured EGFR-mutated NSCLC cells with a combination treatment of icotinib and STAT3 activator IL-6 or STAT3 inhibitor CTN. Compared to single-agent icotinib treatment, the expression of FOXM1 and ATG7 were elevated in the group of icotinib with IL-6. In contrast, CTN remarkably inhibited the activation of STAT3 and abrogated the levels of FOXM1 and ATG7 during icotinib treatment (Fig. 3B). An increase of autophagic flux was observed in the treatment of icotinib and IL-6, and the contrary result was observed in the group of icotinib and CTN (Fig. 3C). When adding IL-6 or CTN to cells treated with icotinib, no significant change but an accumulation of apoptotic cells was respectively detected using Annexin V-PE/7-AAD staining (Fig. 3D). Functionally, IL-6 in icotinib-treated cells exhibited promoted ability to form cell colonies, and CTN in icotinib-treated cells robustly abrogated cell growth (Supplementary Fig. 2A).

Furthermore, the essential role of STAT3 in resistance to chemotherapy and targeted therapy was dependent on its phosphor-Y705 residue. Replacement of tyrosine 705 with phenylalanine facilitated inactivating STAT3 in cancer cells [13]. On the contrary, the substitution of amino acids 662 and 664 to cysteines contributed to STAT3(Y705) constitutive activation [7]. We generated PC-9/GR cells and H1975 cells that expressed wild STAT3 (STAT3WT), dominant negative STAT3 (STAT3-Y705F), and constitutively active STAT3 (STAT3-CA) to analyze the role of STAT3(Y705) in icotinib-induced autophagy. In contrast to the decreased levels of p-STAT3, FOXM1, and ATG7 in cells with the treatment of icotinib and STAT3-Y705F, STAT3-CA constitutively enhanced the expression of p-STAT3, FOXM1, and ATG7 in cells exposed to icotinib (Fig. 3E). As expected, STAT3-Y705F decreased autophagy flux in the cytoplasm of PC-9/GR cells cultured with icotinib, and STAT3-CA displayed the opposite result (Fig. 3F). Notably, cells transfected with STAT3-Y705F during icotinib treatment showed a higher apoptosis rate than the combination of icotinib and STAT3WT group. No significant differences were illustrated in the group of STAT3-CA combined with icotinib treatment (Fig. 3G).

### FOXM1 is defined as a connection between STAT3 and ATG7-dependent autophagy

**Fig. 4:**
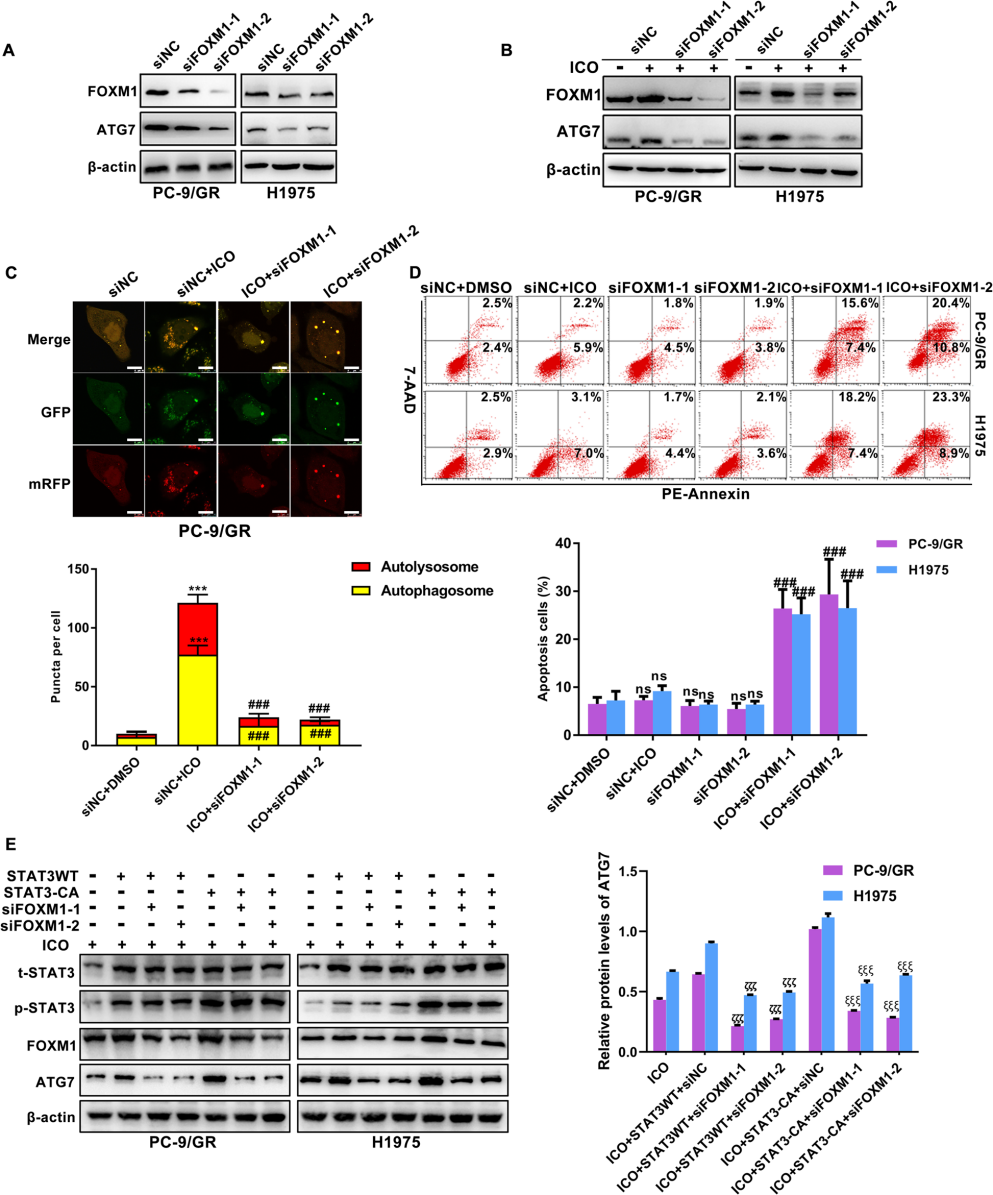
STAT3/FOXM1/ATG7 signalling is essential for icotinib resistance. **A** Immunoblotting was performed to determine the impact of FOXM1 blockade on protein expression of ATG7. **B**-**D** Representative results of protein changes of FOXM1 and ATG7 levels (**B**), autophagic flux with the mRFP-GFP-LC3 reporter (**C**), and cell apoptosis (**D**) in icotinib-treated cells in response to FOXM1 knockdown with two specific siRNAs against FOXM1. **E** Expression of ATG7 was detected in icotinib-treated cells transfected with STAT3-CA and siFOXM1 alone or combination. [***P < 0.001, ns, no significance as compared with the DMSO group. ###*P* < 0.001 as compared with the icotinib group. ζζζ*P* < 0.001 as compared with icotinib plus STAT3WT and siNC. ξξξ*P* < 0.001 as compared with icotinib plus STAT3-Y705F and siNC]

To exclude an off-target impact of FOXM1 on ATG7 expression, we ablated the expression of FOXM1 by using a siRNA approach. As expected, ATG7 was accordingly decreased upon FOXM1 knockdown (Fig. 4A). The addition of icotinib with siFOXM1 had a much more inhibitory effect on ATG7 level than icotinib alone (Fig. 4B). Autophagy flux was reduced in the presence of icotinib and siFOXM1 (Fig. 4C). To extend these findings, we examined cell apoptosis in the combination of icotinib and siFOXM1. A synergistic pro-apoptotic effect was shown when cells were cultured with both icotinib and siFOXM1 (Fig. 4D). Figure 4E shows that up-regulated ATG7 induced by icotinib was impaired by siFOXM1 in cells transfected with STAT3-CA. Taken together, these findings support that activated STAT3/FOXM1 signalling promotes ATG7-dependent autophagy in icotinib-resistant cells.

### Chloroquine and STAT3/FOXM1 signalling overcome resistance to icotinib in vivo

**Fig. 5:**
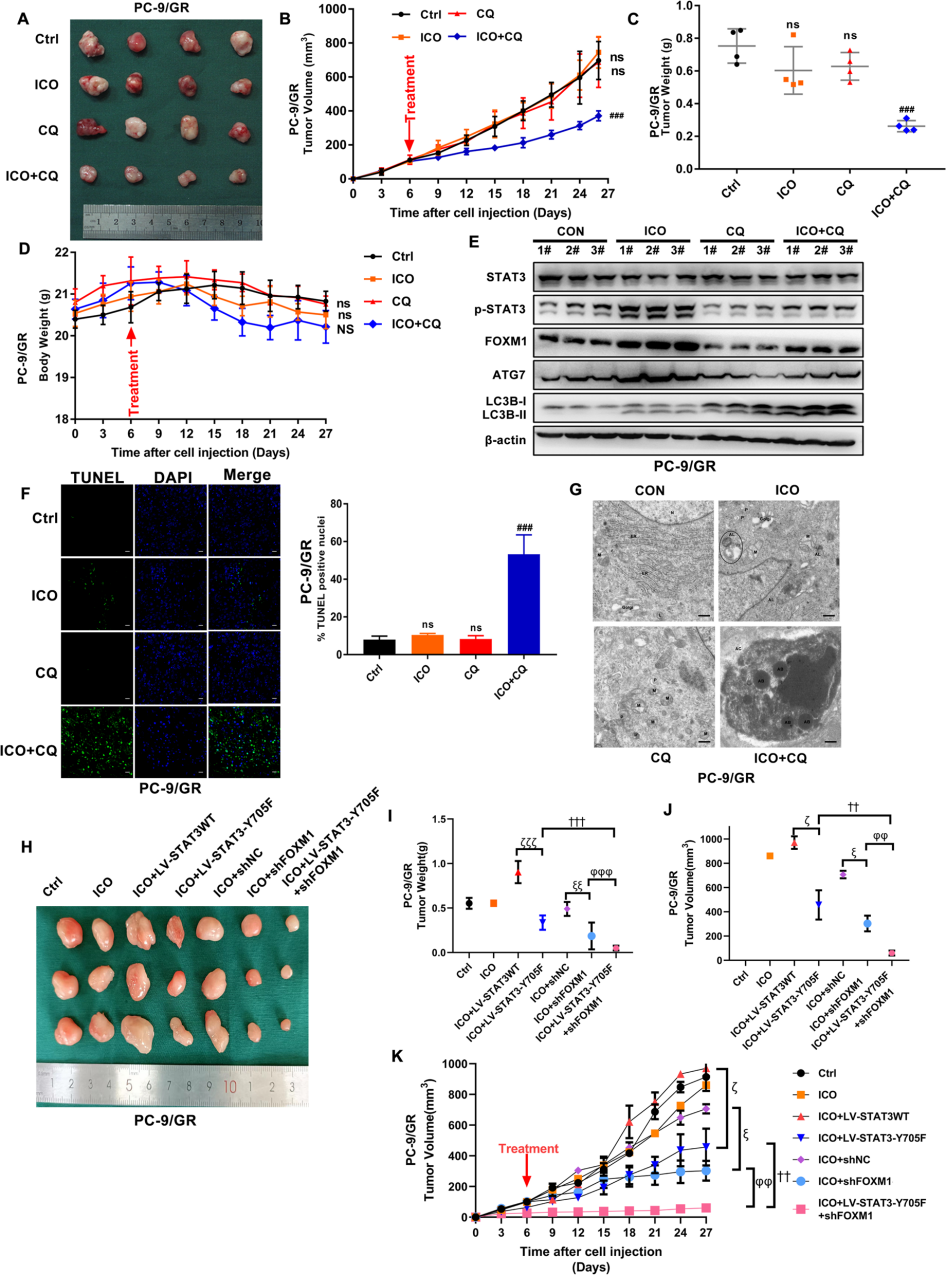
Chloroquine and STAT3/FOXM1 signalling blockade effectively sensitize EGFR-mutated NSCLC cells to icotinib in vivo. **A** Tumours were excised and collected after treatment with icotinib/CQ alone or the combination for 21 days. **B**-**D** Tumour weight (**B**), tumour volume (**C**), and body weight (**D**) of indicated xenografts are shown. **E** Immunoblotting assays measured LC3 conversion and the expression of STAT3, FOXM1, and ATG7. **F** Paraffin-embedded sections of tumour tissues from xenografted mice were analyzed by TUNEL staining. Scale bar: 10 μm. **G** Ultrastructural changes of autophagic vesicles and apoptotic bodies in tumour tissues of indicated xenografts are shown. Scale bar: 1 μm. P, phagosome. AP, autophagosome. AL, autolysosome. AC, apoptotic cell. AB, apoptotic body. ER, endoplasmic reticulum. M, mitochondrion. Golgi, golgi apparatus. **H** Xenograft tumours were excised from the group of icotinib, LV-STAT3-Y705F and shFOXM1 alone or combination. **I**-**K**) Tumour weight (**I**), tumour volume (**J**), and growth curve (**K**) of indicated xenografts are shown. [**P < 0.01, ***P < 0.001, ns, no significance as compared with the control group. #*P* < 0.05, ###*P* < 0.001 as compared with the icotinib alone group. ζ*P* < 0.05, ζζζ*P* < 0.001 as compared with icotinib plus LV-STAT3WT, ξ*P* < 0.05, ξξ*P* < 0.01 as compared with icotinib plus shNC. ††*P* < 0.01, †††*P* < 0.001 as compared with icotinib plus LV-STAT3-Y705F. φφ*P* < 0.01, φφφ*P* < 0.001 as compared with icotinib plus shFOXM1]

Furthermore, we verified the impacts of chloroquine on resistance to icotinib in vivo. Figure 5A displays a marked growth inhibition in icotinib/chloroquine combination, but no effective response was seen with icotinib or chloroquine as monotherapy. Similar results were also presented in tumour weight and volume (Fig. 5B and C). Additionally, the side-effects of icotinib in combination with chloroquine were evaluated via body weight of mice. Figure 5D prompts that no evident side effects were found in either icotinib or chloroquine as a single or a combination. Consistent with the results of HCQ in vitro, WB assay suggested that icotinib induced upregulation of p-STAT3, FOXM1, ATG7, and LC3B-II, but contrary results were claimed in the combination group compared to the single-agent icotinib treatment (Fig. 5E). A statistically increasing apoptosis induced by icotinib in the combination of chloroquine was revealed by TUNEL staining (Fig. 5F). Accordingly, TEM affirmed that apoptotic cells and apoptotic bodies in tissues derived from icotinib/chloroquine combination treated-mice (Fig. 5G). Furthermore, STAT3-Y705F and shFOXM1 lentivirus were used to verify the impacts of STAT3/FOXM1 signalling on resistance to icotinib in xenograft mouse models. We found that STAT3-Y705F and shFOXM1 alone or in combination could inhibit the growth of icotinib-resistant xenograft tumours (Fig. 5H). The weight and volume of tumour were displayed in Fig. 5I-K. These data indicated that autophagy blockade by chloroquine or STAT3/FOXM1 signalling sensitized EGFR mutated NSCLC cells to icotinib in vivo.

### STAT3/FOXM1/ATG7 signalling is associated with therapeutic efficacy of EGFR-TKIs in lung adenocarcinoma patients

**Fig. 6:**
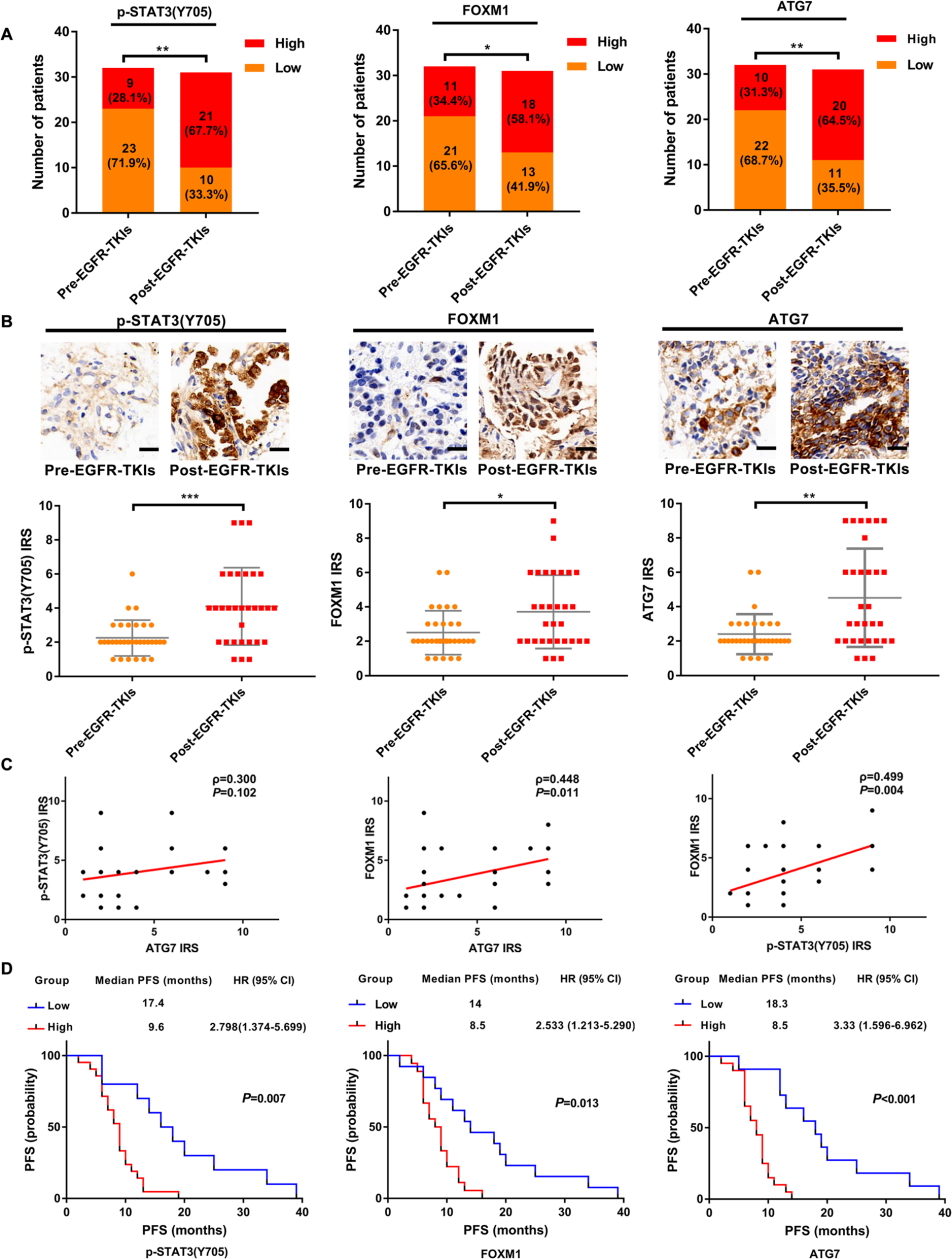
Clinical significance of STAT3/FOXM1/ATG7 signalling in response of EGFR-mutated NSCLC patients to EGFR-TKIs. **A** The proportion of high and low p-STAT3 (Y705), FOXM1, and ATG7 expression in EGFR-TKIs-sensitive (*n* = 32) and EGFR-TKIs-resistant (*n* = 31) patients. **B** Representative images of immunohistochemical staining for phosphorylated STAT3(Y705), FOXM1 and ATG7 in the tumour tissues from EGFR-TKIs-sensitive and EGFR-TKIs-resistant patients. Statistical analysis of IRS of the indicated proteins in each group is depicted. Scale bars, 20 μm. **C** The correlation of active STAT3, FOXM1, and ATG7 in EGFR-TKIs-resistant patients harbouring EGFR mutation. **D** Kaplan–Meier analysis of PFS in resistant patients after EGFR-TKIs treatment (*n* = 31) further stratified with low and high expression of phosphorylated STAT3(Y705), FOXM1 and ATG7 levels. [*P < 0.01, **P < 0.01, ***P < 0.001 as compared with the pre-EGFR-TKIs group]

To extend these in vitro and in vivo findings, we used clinical specimens to further verify the association between EGFR-TKIs resistance and STAT3/FOXM1/ATG7 signalling. Supplementary Table 1 demonstrates patient demographics of the pre-EGFR-TKIs treatment and post-EGFR-TKIs treatment groups. As expected, the percentage of high expression of ATG7 in the post-EGFR-TKIs treatment group was higher than that in the pre-EGFR-TKIs treatment group. Similarly, patients with increased expression of p-STAT3 and FOXM1 trended towards appearing in the post-EGFR-TKIs treatment group (Fig. 6A). In addition, IHC staining detected significantly increased ATG7, p-STAT3 and FOXM1 in the post-EGFR-TKIs treatment group (Fig. 6B). Protein expression of ATG7 was positively correlated with FOXM1 in the post-EGFR-TKIs treatment group. A strong positive correlation was also observed between levels of p-STAT3(Y705F) and FOXM1 in this group. However, no significant correlation was shown between ATG7 expression and p-STAT3 expression (Fig. 6C). Further Kaplan-Meier analyses showed that EGFR-TKIs-resistant patients with high p-STAT3, FOXM1 or ATG7 expression exhibited significantly longer median PFS and median OS than patients with low expression of these proteins (Fig. 6D and Supplementary Fig. 3A). Multivariate Cox regression analysis confirmed that FOXM1 and ATG7 expression negatively correlated with PFS in resistant patients after EGFR-TKIs treatment (Supplementary Table 2). Our findings affirm that there may be a link among STAT3/FOXM1/ATG7 signalling, autophagy and EGFR-TKIs efficacy in EGFR-mutated NSCLC patients.

## Discussion

As documented, protective autophagy is regarded to deliver damaged organelles and misfolded proteins to the lysosome for degradation and readily produces metabolites to maintain cellular homeostasis that can result in tumorigenesis and resistance to treatment [14]. Protective autophagy limited the therapeutic effects of EGFR-TKIs in different cancer cells. Experimentally altering specific EGFR-related genes and pathways caused autophagic cell death or growth [15], suggesting the crucial role of autophagy in the sensitivity and resistance of cancer cells to EGFR-TKIs [16]. Notably, STAT3/FOXM1/ATG7 signalling played a vital supportive role in EGFR-mutated NSCLC resistance to icotinib through inducing aberrant autophagy. Impressively, icotinib could upregulate ATG7 to induce protective autophagy in resistant NSCLC cells, but silencing Beclin-1 or blockade of the class III PI3K complex could not affect protective autophagy initiated by icotinib.

Notably, there is a discrepancy in the biological effects of autophagy induced by icotinib in various NSCLC cells. The pro-apoptotic effect of autophagy induced by icotinib has been reported in HCC827 cells, and it has also been shown that autophagy may not be essential for the resistance of A549 cells to icotinib [17]. However, we found that autophagy could be revealed as a potential therapeutic target for reversing resistance to icotinib in PC-9/GR and H1975 cells. In addition, autophagy indeed relies on distinct mechanisms in different cancer types or tumour treatments. For instance, such a discrepancy of biological effects of Beclin-1 was common, especially in the regulation of autophagy. Beclin-1 was known for its ability to affect the sensitivity of cancer to targeted therapy such as gefitinib, osimertinib and sorafenib [18–20], but was not involved in autophagy-dependent resistance to erlotinib in tongue squamous carcinoma [21]. Similarly, our results displayed that autophagy-modulated resistance to icotinib was independent of Beclin-1. These results indicate a non-essential role for Beclin-1 in autophagy-mediated resistance to targeted therapy. On the other hand, the dual role of ATG7 in lung cancer is context-dependent, including pro-apoptotic and anti-apoptotic effects. For example, ATG7-dependent autophagy suppressed the progression of lung cancer driven by activation of oncogenic HRasV12 [22]. In contrast to the tumour suppressive effect of ATG7 on tumour development [23], blockage by ATG7 deficiency contributed to promoting the sensitivity to chemotherapy in resistant cells [24]. Interestingly, we found that silencing ATG7 also accelerated lung tumour apoptosis induced by icotinib, suggesting the importance of ATG7 in autophagy-dependent resistance to icotinib.

Activation of STAT3 is a well-known event in resistance to therapy. For instance, activated STAT3 was believed to be necessary for paclitaxel-induced autophagy due to the addition of IL-6 [25], but acted to inhibit docetaxel-regulated autophagy in castration-resistant prostate cancer cells [26], leaving the puzzle of how STAT3 affecting autophagy unresolved. We proposed that activation of STAT3 was capable of elevating FOXM1 expression to promote ATG7-dependent autophagy, leading to resistance to icotinib treatment. Until now, FOXM1 was reported to increase the expression of LC3 and Beclin-1 by binding to their promoters [27]. We indicated that ATG7 could be a functional target of FOXM1 in autophagy initiated by icotinib. Our findings established a connection between STAT3/FOXM1 signalling and ATG7-dependent autophagy to maintain icotinib resistance in EGFR-mutated NSCLC.

## Conclusion

In summary, we confirmed that STAT3/FOXM1/ATG7 signalling might be essential for autophagy induced by icotinib in resistant lung cancer cells. We also demonstrated that ATG7 blockade promoted cellular toxicity of icotnib in resistance cells, providing new insights into the clinical applications of autophagy inhibitors to reverse icotinib resistance. Additional researches are needed to be conducted and will hopefully result in observations of clinical relevance. For example, pending further clinical data of icotinib/STAT3 inhibitors or icotinib/chloroquine therapeutic combinations might be characterized as a novel approach for overcoming the resistance to icotinib.

## Supplementary Information

**Additional file 1.**

## Data Availability

All data generated or analysed during this study are included in this manuscript and its supplementary information files.

## Abbreviations

NSCLC: non-small cell lung cancer
EGFR-TKIs: Epidermal growth factor receptor-tyrosine kinase inhibitors
PARP: poly (ADP-ribose) polymerase
CQ: chloroquine
BafA1: bafamycin A1
ATG3: autophagy related 3
ATG5: autophagy related 5
ATG7: autophagy related 7
ATG14: autophagy related 14
STAT3: signal transducer and activator of transcription 3
FOXM1: forkhead box M1
3MA: 3-methyladenine
CCK-8: Cell Counting Kit-8
DAPI: 40,6-diamidino-2-phenylindole dihydrochloride
TEM: transmission electron microscopy
IL-6: interleukin-6
CTN: cryptotanshinone
DMSO: dimethyl sulfoxide
LC3: microtubule-associated protein 1A/ 1B-light chain 3
mTOR: mammalian target of rapamycin
PFS: progression-free survival
OS: overall survival
