# Supplementary material for: Essential role for STAT3/FOXM1/ATG7 signaling-dependent autophagy in resistance to Icotinib
Source: J Exp Clin Cancer Res. 2022 Jun 11;41:200. doi: 10.1186/s13046-022-02390-6 (PMC9188165; doi:10.1186/s13046-022-02390-6)
Supplement: Supplementary file 1 — Additional file 1. [file 13046_2022_2390_MOESM1_ESM.zip › Supplementary Figure Legends.docx]

**Supplementary Figure Legends**

**Supplementary Figure 1 Class III PI3K complex-independent of autophagy induced cell resistance to icotinib.** Representative images and quantification of cell colonies treated by icotinib in the absence and presence of autophagy inhibitor CQ, BafA1, and 3-MA treatment. [ns, no significance as compared with the DMSO group. ###P < 0.001, NS, no significance as compared with the icotinib group.]

**Supplementary Figure 2 The pro-proliferative impact of STAT3 activity on icotinib-resistant cells.** Representative images and quantification of cell colonies treated by icotinib in the absence and presence of STAT3 activator IL-6 and inhibitor CTN treatment. [ns, no significance as compared with DMSO group. ###P < 0.001 as compared with icotinib alone group.]

**Supplementary Figure 3 Clinical associations between STAT3/FOXM1/ATG7 signalling and OS in resistant patients after EGFR-TKIs treatment.** Kaplan–Meier analysis of OS in resistant patients after EGFR-TKIs treatment (n = 31) further stratified with low and high expression of phosphorylated STAT3(Y705), FOXM1 and ATG7 levels.

**Supplementary Table1 Clinical and molecular characteristics**

|  |  | Pre-EGFR-TKIs  N (%) | Post-EGFR-TKIs  N (%) |
| --- | --- | --- | --- |
| Sex | Male | 15(46.9%) | 11(35.5%) |
|  | Female | 17(53.1%) | 20(64.5%) |
| Age（Years） | ≤61^#^ | 17(53.1%) | 16(51.6%) |
|  | ＞61 | 15(46.9%) | 15(48.4%) |
| Smoking history | Yes | 13(40.6%) | 10(32.3%) |
|  | No | 19(59.4%) | 21(67.7%) |
| Tumour size(cm) | ≤4 | 10(31.3%) | 12(38.7%) |
|  | ＞4 | 22(68.7%) | 19(61.3%) |
| TNM stage | Ⅲ | 9(28.1%) | 10(32.3%) |
|  | Ⅳ | 23(71.9%) | 21(67.7%) |
| Pretreatment EGFR mutation | Exon 19 deletion | 20(62.5%) | 19(61.3%) |
|  | Exon 21 L858R | 12(37.5%) | 12(38.7%) |
| Therapy | Gefitinib | 19(59.4%) | 17(54.8%) |
|  | Icotinib | 13(40.6%) | 14(45.2%) |
| ATG7 | low | 22(68.7%) | 11(35.5%) |
|  | high | 10(31.3%) | 20(64.5%) |
| p-STAT3 | low | 23(71.9%) | 10(32.3%) |
|  | high | 9(28.1%) | 21(67.7%) |
| FOXM1 | low | 21(65.6%) | 13(41.9%) |
|  | high | 11(34.4%) | 18(58.1%) |

#Median

**Supplementary** **Table2 Univariate and multivariate analyses of variables associated with PFS**

| Characteristics | | PFS | | | | | |
| --- | --- | --- | --- | --- | --- | --- | --- |
|  |  | Univariate Cox | | | Mulvariate Cox | | |
|  |  | HR | *P* | 95% CI | HR | *P* | 95% CI |
| Sex |  |  |  |  |  |  |  |
|  | Male | 1.000 |  |  | 1.000 |  |  |
|  | Female | 0.364 | 0.018 | 0.158-0.842 | 0.733 | 0.615 | 0.219-2.456 |
| Age（years） |  |  |  |  |  |  |  |
|  | ≤61**^#^** | 1.000 |  |  |  |  |  |
|  | **＞**61 | 0.698 | 0.355 | 0.326-1.496 |  |  |  |
| Smoking |  |  |  |  |  |  |  |
|  | Yes | 1.000 |  |  | 1.000 |  |  |
|  | No | 0.279 | 0.004 | 0.117-0.668 | 0.615 | 0.436 | 0.181-2.089 |
| Tumour size（cm） |  |  |  |  |  |  |  |
|  | ＜5 | 1.000 |  |  |  |  |  |
|  | ≥5 | 1.331 | 0.485 | 0.596-2.976 |  |  |  |
| TNM stage |  |  |  |  |  |  |  |
|  | Ⅲ | 1.000 |  |  |  |  |  |
|  | Ⅳ | 1.348 | 0.469 | 0.601-3.021 |  |  |  |
| Pretreatment EGFR mutation |  |  |  |  |  |  |  |
|  | Exon 19 deletion | 1.000 |  |  | 1.000 |  |  |
|  | Exon 21 L858R | 3.243 | 0.009 | 1.342-7.822 | 1.488 | 0.452 | 0.528-4.197 |
| Therapy |  |  |  |  |  |  |  |
|  | Gefitinib | 1.000 |  |  |  |  |  |
|  | Icotinib | 0.618 | 0.206 | 0.293-1.303 |  |  |  |
| ATG7 |  |  |  |  |  |  |  |
|  | low | 1.000 |  |  | 1.000 |  |  |
|  | high | 7.977 | ＜0.001 | 2.555-24.907 | 9.041 | 0.002 | 2.203-37.097 |
| p-STAT3 |  |  |  |  |  |  |  |
|  | low | 1.000 |  |  | 1.000 |  |  |
|  | high | 4.502 | 0.003 | 1.692-11.976 | 1.560 | 0.493 | 0.437-5.563 |
| FOXM1 |  |  |  |  |  |  |  |
|  | low | 1.000 |  |  | 1.000 |  |  |
|  | high | 3.755 | 0.005 | 1.494-9.439 | 4.599 | 0.010 | 1.441-14.679 |

Abbreviation: PFS, progression-free survival. CI, confidence interval.

#Median. *HRs were derived from univariable and multivariable Cox proportional hazards regression analysis, and models were adjusted for all confounding factors listed in the table.
