# Supplementary figures and images for: Essential role for STAT3/FOXM1/ATG7 signaling-dependent autophagy in resistance to Icotinib
Source: J Exp Clin Cancer Res. 2022 Jun 11;41:200. doi: 10.1186/s13046-022-02390-6 (PMC9188165; doi:10.1186/s13046-022-02390-6)

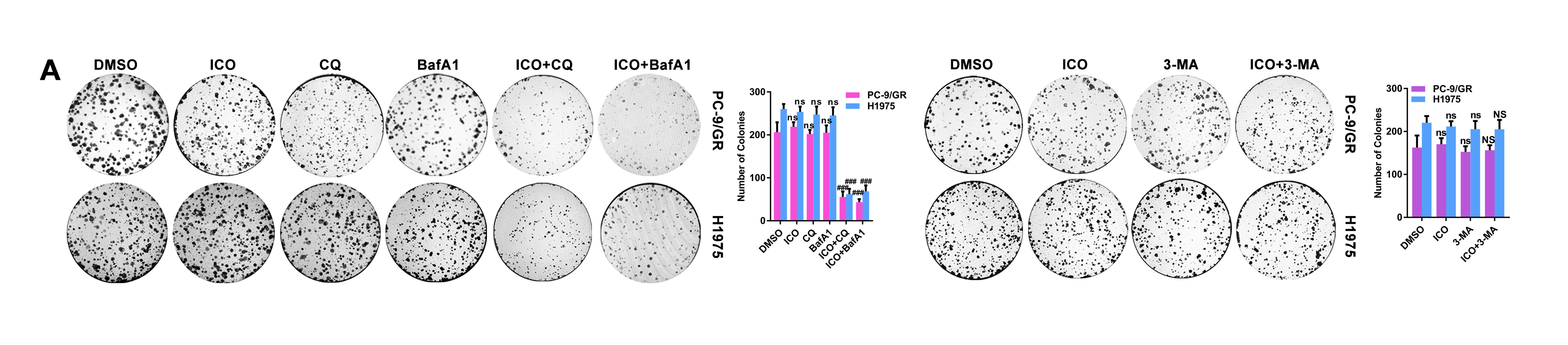

Supplement: Supplementary file 1 — Additional file 1. [file 13046_2022_2390_MOESM1_ESM.zip › SFig 1.tif]

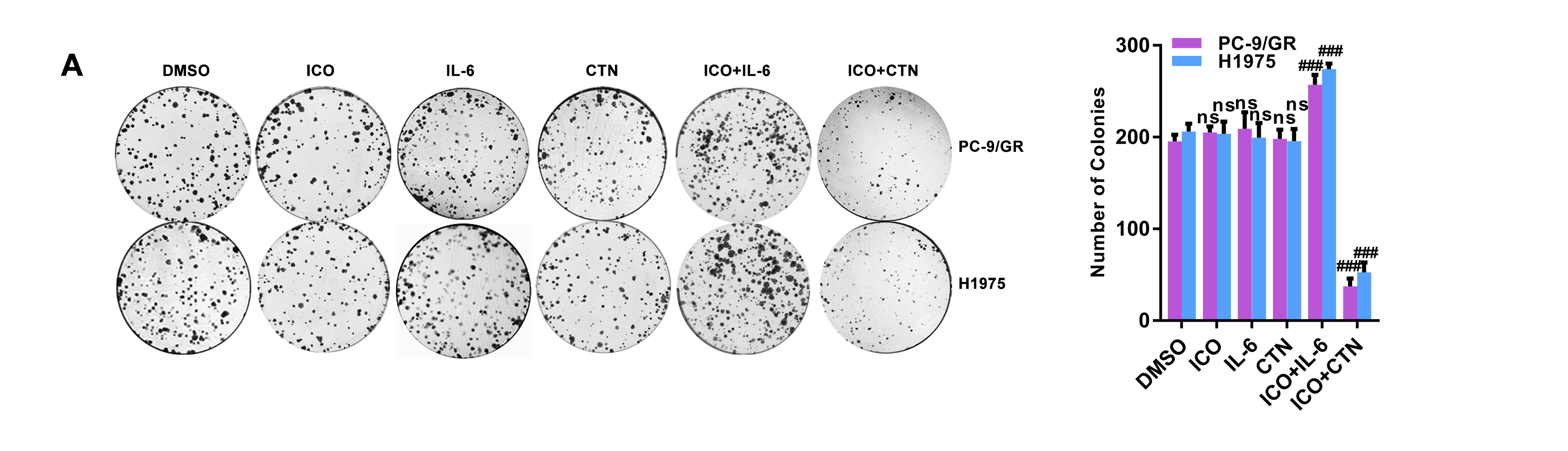

Supplement: Supplementary file 1 — Additional file 1. [file 13046_2022_2390_MOESM1_ESM.zip › SFig 2.tif]

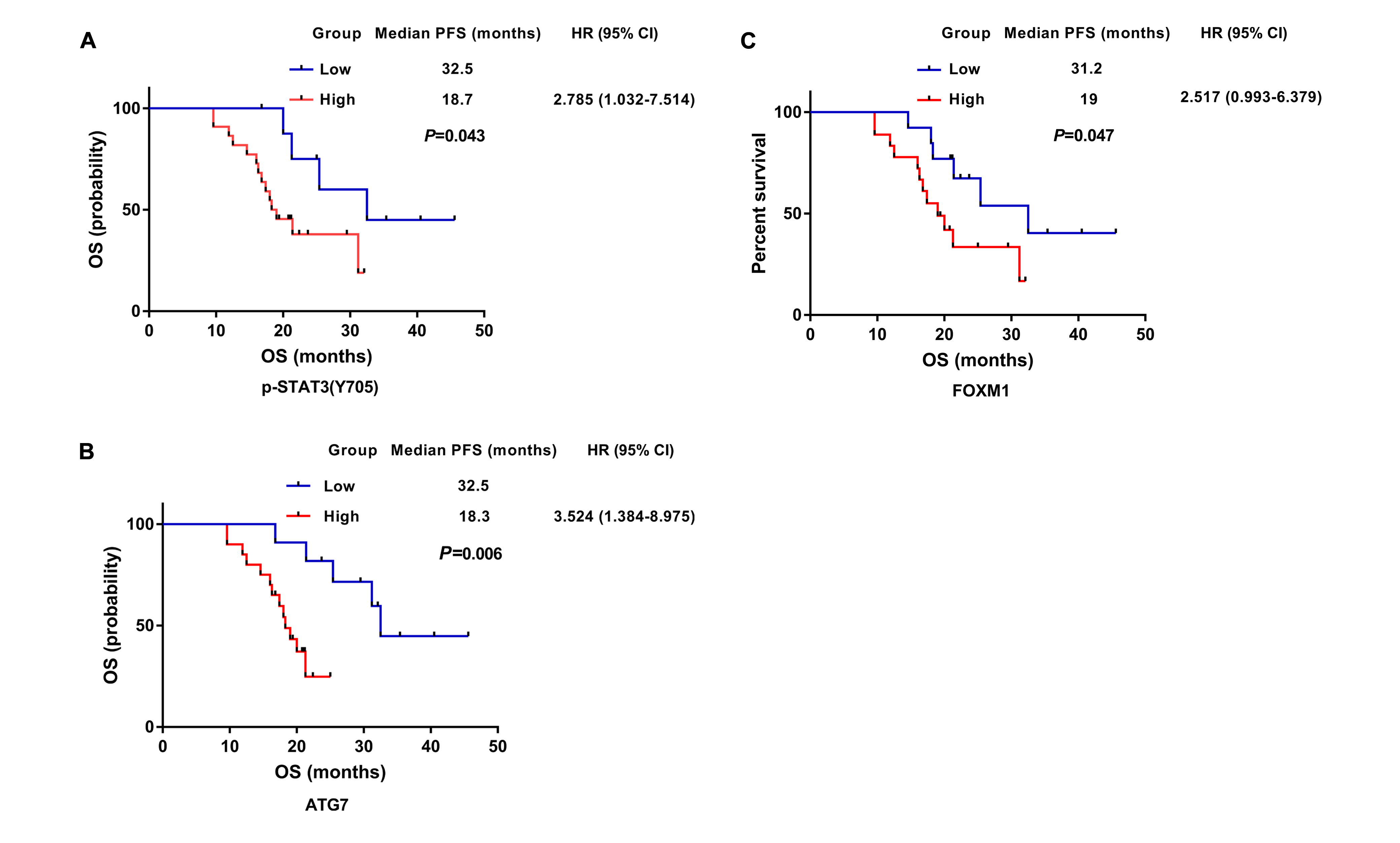

Supplement: Supplementary file 1 — Additional file 1. [file 13046_2022_2390_MOESM1_ESM.zip › SFig 3.tif]
